# Impact of Short Social Training on Prosocial Behaviors: An fMRI Study

**DOI:** 10.3389/fnsys.2016.00060

**Published:** 2016-07-05

**Authors:** Evgeniya Lukinova, Mikhail Myagkov

**Affiliations:** ^1^Skolkovo Institute of Science and Technology, MoscowRussia; ^2^Department of Political Science, University of Oregon, Eugene, ORUSA

**Keywords:** prosocial actions, sociality, value calculation, social cognition, BCI

## Abstract

Efficient brain–computer interfaces (BCIs) are in need of knowledge about the human brain and how it interacts, plays games, and socializes with other brains. A breakthrough can be achieved by revealing the microfoundations of sociality, an additional component of the utility function reflecting the value of contributing to group success derived from social identity. Building upon our previous behavioral work, we conduct a series of functional magnetic resonance imaging (fMRI) experiments (*N* = 10 in the Pilot Study and *N* = 15 in the Main Study) to measure whether and how sociality alters the functional activation of and connectivity between specific systems in the brain. The overarching hypothesis of this study is that sociality, even in a minimal form, serves as a natural mechanism of sustainable cooperation by fostering interaction between brain regions associated with social cognition and those related to value calculation. We use group-based manipulations to induce varying levels of sociality and compare behavior in two social dilemmas: Prisoner’s Dilemma and variations of Ultimatum Game. We find that activation of the right inferior frontal gyrus, a region previously associated with cognitive control and modulation of the valuation system, is correlated with activity in the medial prefrontal cortex (mPFC) to a greater degree when participants make economic decisions in a game with an acquaintance, high sociality condition, compared to a game with a random individual, low sociality condition. These initial results suggest a specific biological mechanism through which sociality facilitates cooperation, fairness and provision of public goods at the cost of individual gain. Future research should examine neural dynamics in the brain during the computation of utility in the context of strategic games that involve social interaction for a larger sample of subjects.

## Introduction

Daily life confronts us with social situations and interactions on a regular basis. Thus, economic decisions are often embedded in a social context. However, we rarely think of how the brain processes the decisions we make, especially those decisions that affect the outcomes of other people with our decisions. Social factors such as group membership and affiliation motives have powerful effects on a range of behaviors, suggesting that these factors carry substantial decision utility for people. However, this “social utility” is rarely included in the formal models of economic behavior. This paper triangulates the theories of human social behavior from social psychology, decision modeling techniques from behavioral economics, and brain-imaging tools from neuroscience to draw a more precise picture of the mechanisms by which social factors influence economic decisions.

Recent efforts to unite these traditions have proven fruitful in delivering theoretical insights and a model-based precision to the study of economic behavior in a realistic social context (Akerlof and Kranton, 2000, 2010; Perc and Szolnoki, 2010; Perc et al., 2013; Lukinova et al., 2014; Berkman et al., 2015). One behavioral study (Berkman et al., 2015) confirmed that socialization induces prosocial behavior in economic games due to sociality. Participants are introduced in groups creating group differentiation, which easily satisfies the minimal group requirement (Tajfel, 1978). In line with Social Identity Theory we assert some esteem or value is gained by boosting the group (or derogating the outgroup; Tajfel and Turner, 1979) and, in turn, this additional value (Lukinova et al., 2014) plays a decisive role in encouraging prosocial actions. Building upon results of our behavioral work, we now report on a series of functional magnetic resonance imaging (fMRI) experiments that utilize the fMRI technology in combination with socialization and well-known economic games to measure whether and how sociality alters the functional activation of and connectivity between the social cognition network and the valuation network in the brain.

We use experiments in fMRI and laboratory facilities in an attempt to study the neural mechanisms of human social interactions and the microfoundations of prosocial behavior. The main novelty and contribution of this paper is in the reality of social interactions as opposed to artificial social interactions and in the combination of two social dilemmas in the fMRI study. To our knowledge the Prisoner’s Dilemma (PD) and various representations (sequential vs. simultaneous; matrix vs. a game tree) of Ultimatum Game (UG) have not appeared together in prior neuroeconomics research. Our study follows pioneer fMRI studies that compare PD to other games (e.g., Stag Hunt; Emonds et al., 2011, 2012). Besides regular UG we use Welfare Game (WG), a novel 2 × 2 simultaneous version of UG that preserves the distributional essence of the game, as well as its antecedent, the UG.

Using fMRI gives us a unique perspective on how sociality works. We examine neural dynamics in specific systems when people compute their utility in the context of strategic games that involve various levels of social interaction. Social science researchers use neuroimaging as the key tool to understand the nature of the various peculiar aspects of human behavior such as “economic irrationality” (Peterson, 2005), altruism and “altruistic punishment” (De Quervain et al., 2004; Waytz et al., 2012), asymmetry between gains and losses (Yacubian et al., 2006), cooperation (Fett et al., 2014; Watanabe et al., 2014), preference of egalitarian outcomes (Sanfey et al., 2003; Tricomi et al., 2010; Dawes et al., 2012; Yamagishi et al., 2012; Osinsky et al., 2013), decision about an unfair split (Güroğlu et al., 2014), and theory of mind (Lee and Harris, 2015; Strombach et al., 2015). If social neuroscience (Cacioppo et al., 2002; Norman et al., 2012) attempts to understand mechanisms that underlie social behavior using a mix of biological and social approaches (Willingham and Dunn, 2003), neuroeconomics opens up the “black box” of the brain by finding neural correlates of choice behavior (Camerer et al., 2005; Lohrenz and Montague, 2008; Glimcher and Fehr, 2013; Lampert et al., 2014; Schroeder and Graziano, 2015) and behavior under risk and uncertainty (Hsu et al., 2005). Unfortunately, current knowledge of neural mechanisms in prosocial decision making is still limited (Fehr and Camerer, 2007; Lee, 2008; Emonds et al., 2011, 2012, 2014; Declerck et al., 2013; Declerck and Boone, 2015; Kuss et al., 2015).

For the purposes of this paper, sociality, or social utility, is defined as an additional component of the utility function reflecting the value of contributing to group success derived from social identity, defined as knowledge, value, and emotional significance for group membership (Tajfel, 1982). In economic terms, social identity may be one of the mechanisms by which sociality comes to have a positive decision utility. There are many ways of manipulating sociality for the purpose of testing its effect on economic decisions and the associated neural systems. To our knowledge, a formal typology of the various kinds of sociality is not currently available, even though such a typology would be quite useful for the present line of research and related efforts. In the course of our behavioral research, we surveyed the relevant literature and identified two broad classes of social manipulations (Low sociality and High sociality). We follow on this distinction in our fMRI study. In particular, during our Pilot fMRI Study we compare playing with humans to playing with computers, whereas in the main study we focus on the difference between the behavior in economic games where the opponent is a random individual or an acquaintance.

We have specific, *a priori* hypotheses about the likely brain regions involved in each of the two target processes (sociality and valuation). A growing body of work implicates the ventral striatal dopamine circuit in the integration and calculation of subjective value or utility, including the ventromedial prefrontal cortex (vmPFC), the ventral aspects of the caudate (vC), and the nucleus accumbens (nAcc; Plassmann et al., 2007; Hare et al., 2008; Ruff and Fehr, 2014). Social cognition, on the other hand reliably recruits activation in a network of brain regions including the dorsomedial prefrontal cortex (dmPFC), the posterior cingulate cortex (PCC), and the temporoparietal junction (TPJ; Amodio and Frith, 2006; Van Overwalle, 2009). Pertinent to the present research, a recent study found that activity in TPJ tracked perceived social distance between an actor and a target, and interacted with the vmPFC, a region involved in value calculation, to modulate the actor’s decisions about how to divide up a fixed pot of money to be shared by the actor and the target (Strombach et al., 2015). This study provides proof-of-concept that social cognition regions can interact with valuation regions to influence economic decisions. Existent reviews in neuroeconomics add another neural network that is consistently recruited when people face social dilemmas, i.e., network related to cognitive control (Declerck et al., 2013). Thus, one can formulate a competing hypothesis: the interaction of cognitive control and valuation regions of the brain facilitate prosocial decision making.

Since our experimental design includes two types of economic games we can also examine the question of whether the neural bases of social welfare choices are different from those of collective action. The regions of the brain associated with reward and valuation are under our focus and we hypothesize that these brain regions should be more active during the fair condition than during the unfair condition in the UG. Indeed, the vmPFC is reported to activate during tasks involving inequality in social settings (Fliessbach et al., 2007; Tabibnia et al., 2008; Tricomi et al., 2010; Aoki et al., 2015). Inequality is noticed by participant once reward comparison between the other and herself is accomplished. However, it also hurts when she realizes that she falls behind. We hypothesize that there will be an increased activity in the reward associated brain regions (vmPFC) as well as the brain regions critical for processing emotions, such as amygdala and OFC (Davidson et al., 2000; Dolan, 2002) in variations of UG condition as opposed to the PD game condition.

A more precise understanding of the mechanisms by which sociality affects economic decisions in a collective action situation is the essential next step in improving social brain–computer interfaces (BCIs; Sexton, 2015).

## Materials and Methods

All participants are recruited through advertisements on campus. All subjects are right handed, healthy, have normal or corrected-to-normal vision, have no history of psychiatric diagnoses, neurological or metabolic illnesses, and are not taking medications that can interfere with the performance of fMRI. Participants can be of any gender and ethnicity, but must be at least 18 years old. The only exclusion criterion is based on MRI safety screening (ferromagnetic metal in the body, e.g., dental braces). The participants in the fMRI experiment can earn $5 just for showing up on the day of experiment and up to $20 more, depending on their decisions throughout the game. During all game conditions the participants earned a number of points that was later transferred to money. Subjects provide written informed consent approved by the University of Oregon Human Studies Committee.

On the day of experiment, four people are invited to the conference room in Lewis Center for neuroimaging (LCNI) that is adjacent to the scanning suite. Thus, in every experiment one participant for the fMRI experiment is paired with three other subjects for the reality of the high sociality conditions: Human and Acquaintance. Before they participate in economic games the subjects have time to get to know each other and engage in an informal conversation, i.e., undergo socialization, the technique adopted from our behavioral research (Berkman et al., 2015). Specifically, the participants are asked to introduce themselves by name to the others and say one exciting thing about themselves. The participants then embark on a 10-min interaction with the goal of creating a list of five attributes they all have in common to report back to the experimenter. Finally, one of the participants is asked to go to the scanning room for fMRI experiment and remaining three participants stay in the conference room and proceed with a computer experiment.

Computer experiment laboratory data (*N* = 75) are collected with the help of the z-Tree (Zurich Toolbox for Readymade Economic Experiments) software package (Fischbacher, 2007). The stimuli presentation is identical to the fMRI experiment, with one row chooser and two column choosers (one of the column choosers plays against a predetermined computer strategy).

### Pilot Study

Subjects of the fMRI experiment are 10 UO college students (five females). Stimuli include two sociality conditions (human and computer opponents), two game conditions (PD and WGs), a feedback screen that shows profit of participant based on her decision, and a control condition.

The PD payoff matrix is formed around (1, 2, 4, 6; **Table 1**) values. When one participant defects and the other cooperates, then defector gets the maximum value – 6, and the cooperator receives the minimum payoff of 1. If both cooperate, participants get four each, whereas if both defect, they get two each.

**Table 1:** Prisoner’s dilemma (PD) and Welfare game (WG) payoffs.

	**PD**				**Welfare Game**	
	**L**	**R**			**L**	**R**
		
U	4, 4	1, 6		U	2, 6	6, 2
D	6, 1	2, 2		D	1, 1	4, 4

*PD and WG payoffs are presented in the matrix form. One of the players chooses between rows, i.e., between up (U) and down (D). The other player (randomly paired) chooses between columns, i.e., between left (L) and right (R).*

The WG is a novel game not seen in prior research. It resembles a simultaneous version of the UG with an option for an unfair offer. The UG is a game often played in laboratory experiments in which two players interact to decide how to divide a sum of money that is given to them. One of the players proposes how to divide the sum between the two players, and the other can either accept or reject this proposal. If the second player rejects the proposal, none of the players receive anything. However, if the second player accepts, the money is split according to the proposal. Usually the game is played only once or with a randomly chosen partner so that reciprocation is not an issue. For the same reason, players do not change roles within one game. The equilibrium in the UG is not in the favor of the second player. By rejecting the proposal, the second is choosing nothing rather than something. So, for a rational player it would be better to accept any proposal that gives any amount bigger than 0. Contrary to the economic theory of self-interest, multiple studies (Henrich, 2004; Oosterbeek et al., 2004) show that in many cultures people offer (50:50) splits and offers less than 20% are usually rejected.

The WG’s payoff structure corresponds to values in PD (1, 2, 4, 6; **Table 1**) Based on the payoffs the row chooser always prefers to choose up. The column chooser’s best response to the row chooser’s dominant strategy is to choose left. That is why the Nash equilibrium is (2; 6). However, the row chooser always gets a worse payoff than the column chooser. So, if the row chooser prefers egalitarian outcomes, the row player’s deviation from the equilibrium can occur and result in either of the two egalitarian options: (1; 1) and (4; 4).

Prior to entering the scanner the subjects complete a series of practice trials of a similar game on paper. This ensures that the participants understand and are ready for the stimuli presented in the actual experiment. Participants are told on the day of experiment that they will be Row choosers [choosing between up (U) and down (D)] and will maintain the same role for the whole experiment. Subjects are instructed to look at the central plus sign, and had to switch their attention from the central plus sign to the game stimulus (a table 2 × 2 that is centered on the central plus sign) in each trial to determine the their response by pressing either left or right button on the button box in their right hand. Subjects know that by pressing the left button, they choose up (U) and by pressing the right button – down (D). This study uses deception. Participants are told that their opponents in the high sociality condition are humans. In reality, the participant in the fMRI study always plays a computerized strategy with fixed probabilities: for PD game, right (R) with *p* = 0.85, left (L) with *p* = 0.15; for WG, L with *p* = 0.85, R with *p* = 0.15. Feedback collected following the experiment indicates that the deception was effective and that subjects believed that their opponents were human.

In order to answer the research questions the following neural experimental design is used. The experiment consisted of four blocks [Humans + PD (PG1), Humans + WG (PG2), Computers + PD (CG1), Computers + WG (CG2)] with events within each block. To distinguish between blocks the instruction screen in the beginning of each block indicates whether the participant will play a computer or a human. The game condition does not change throughout the block. The blocks are alternated: for half of the participants the order is PG1, PG2, CG1, CG2, for the other half – CG1, CG2, PG1, PG2. We use an event-related fMRI design with a pseudorandom (predetermined unpredictable) order of game and control condition within a block with the same interstimulus and intertrial intervals used in M. Posner attention studies (Flombaum and Posner, 2005; Abdullaev et al., 2010) that approximate an exponential distribution with a certain mean. The jittering of the time intervals between game and feedback and between feedback and the next trial is done in order to separate brain activity to the game and feedback stimuli.

**FIGURE 1:**
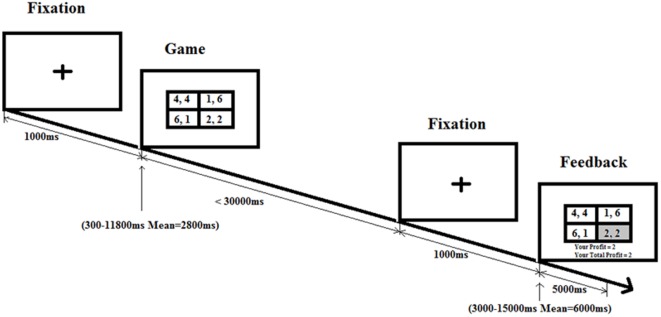
**Game condition schedule.** Following the fixation cross, there is a game phase for each trial, where participants need to make a choice in a social dilemma. After pressing on the button box, participants are shown a feedback screen displaying the profit the participants earn based on their decision and the decision of their partner.

In the game condition (**Figure 1**), the plus sign remains on the center of the screen for 1000 ms. The game stimulus follows after a variable interval (“one of 12 predetermined intervals including three 300 ms intervals, and one each of 550, 800, 1050, 1550, 2300, 3300, 4800, 6550, and 11800 ms, approximating an exponential distribution with a mean interval of 2800 ms;” Abdullaev et al., 2010). The game stimulus stays until response or for 30000 ms. Then a fixation screen is on for 1000 ms followed by another variable intertrial interval (mean of 6000 ms) and finally the feedback screen is on for 2000 ms till the onset of the next trial.

In the control condition, the plus sign remains on the center of the screen for 1000 ms. The control stimulus (each cell in **Figure 1** 2 × 2 table is replaced with “X, X”) follows after a variable interval (mean of 2800 ms). The control stimulus stays until response or for 5000 ms. As in the game condition, then fixation screen is on for 1000 ms followed by another variable intertrial interval (mean of 6000 ms) till the onset of the next trial. Four blocks are presented, and each block has 30 trials (20 game condition trials and 10 control condition trials) with a different pseudorandom order of conditions and intervals.

Responses are recorded with two buttons on an MRI-compatible button box. Reaction times (RT) are measured from the game stimulus to the button press. The control trial is constructed in order to isolate the mechanical activity of the finger pressing on the button box. We expected that with each button press, we should see the ipsilateral cerebellum and the contralateral primary motor cortex activation. Also there is a 30 s baseline period in front of each block with no stimuli except a central plus sign for fixation. So that each condition of the task can be compared to the baseline period.

fMRI stimuli are presented for the participant in the MRI scanner and behavioral data are collected using the Presentation program^1^ run on a computer. Stimuli are presented with a digital projector/reverse screen display system to the screen at the back end of the MRI scanner bore. Subjects see the screen via a small tilted mirror attached to the birdcage coil in front of their eyes.

Imaging is performed using a 3T Siemens Allegra head-only MRI scanner at Lewis Center for Neuroimaging. A standard birdcage coil is used to acquire data from the entire brain. Subjects wear earplugs and earphones to protect their hearing. Additional soft padding is used between earphones and inside the wall of the head coil to diminish head movements.

For functional MRI, the EP2D-BOLD (Blood oxygen level dependent) sequence is run with repetition time (TR) = 2000 ms, echo time (TE) = 30 ms, flip angle = 90°, Field of View (FOV) = 200 mm. The brain is covered with 32 4 mm thick slices acquired in a custom manner (first even slices and then odd slices). For structural MRI scan, the 3D Magnetization Prepared Rapid Acquisition Gradient Echo (MPRAGE) TR = 2500 ms, TE = 4.3 8ms, flip angle = 8°, FOV = 256, 160 slices is run for 8 min to acquire 1 mm^3^ high resolution anatomical scans for registration purposes.

### Main Study

Subjects of the fMRI experiment are 15 individuals recruited from the Eugene, OR community, college-aged (eight females). The procedures are nearly identical to the Pilot Study, except for the sociality, game conditions, and fMRI acquisition. Stimuli include two within-subject opponent conditions, Low sociality (Random Individual), and High sociality (Acquaintance), and two within-subject game conditions, PD, UG as responder. Thus, each trial falls into one of four cells, with participants playing anonymously against either someone from the Eugene community who the participant has not met or someone from the socialized group, and playing the PD game, or the UG game as responder. This study uses deception. Although participants believe they are playing with real people (according to participants’ feedback), the opponent in all three games is in reality a computer that follows the Nash equilibrium strategy with random noise to reduce suspicion. Following the game phase of each trial, participants are shown a feedback screen displaying the profit the participant earned based on their decision and the decision of the partner.

Therefore, the experiment consists of four blocks (Acquaintance + PD, Acquaintance + Ultimatum, Random Individual + PD, Random Individual + Ultimatum) with 30 trials within each block (20 experimental and 10 control trials). To distinguish between blocks the instruction screen in the beginning of each block specifies, whether the participant would play against a person from his/her Socialized group (Acquaintance) or against a Random Individual.

The PD game payoffs are the same as in Pilot Study (**Table 1**). The UG is modeled in such a way that a participant always chooses between two options, either accept an unfair offer that corresponds to up (U) or reject the offer pressing down (D). Equally likely are offered (2; 6) and (3; 5) splits, where the lesser value is an offer to a participant.

MR scans are acquired in the Siemens Skyra 3 Tesla scanner at LCNI, a research-dedicated, whole-body system optimized for functional brain imaging. Participants are situated in the scanner by one of LCNI’s imaging technicians, who also control the scanner during the session. Experimental stimuli (e.g., images, instructions) are presented using a magnet-compatible, rear-projection system controlled by a PC using Presentation Software. Participant responses (e.g., up/down decisions) are collected on a 10-key button box (only two buttons are used) capable of recording responses to the millisecond level. A shimming protocol maximizes homogeneity in the field, and a 30 s, T2*-weighted scout allows slice prescriptions for all subsequent scans. We acquire a high-resolution anatomical T1-weighted MP-RAGE scan (TR/TE = 2300/2.1 ms, 192 × 192 matrix, 1 mm thick, 160 sagittal slices, FOV = 256), functional images with a T2*-weighted echo-planar scan (33 axial slices, TR/TE = 2000/30 ms, 90-deg flip, 64 × 64 matrix, 4 mm thick, FOV = 200), and in-plane gradient echo field map magnitude and phase images to correct for inhomogeneities in the magnetic field (33 axial slices, TR/TE = 345/8.06 ms, 40-deg flip, 64 × 64 matrix, 4 mm thick, FOV = 200).

### Analysis

The epochs used for the analysis are from the game stimulus on-set until response.

#### FSL Procedures

The Pilot Study is first analyzed using General Linear Modeling (GLM) as implemented in the FSL 5.0.2 (FMRIB Software Library). fMRI data is analyzed using FEAT (FMRIB Expert Analysis Tool) available as part of FSL^2^ (Smith et al., 2004). Preprocessing includes the default options, such as separating images of brain from the rest of the images of the head, i.e., creating a brain mask, using the Brain Extraction Tool (BET; Smith, 2002), pre-whitening for local autocorrelation correction using FILM (FMRIB Improved Linear Model; Woolrich et al., 2001), motion correction based on rigid-body transformations using MCFLIRT (Motion Correction FMRIB Linear Image Registration Tool; Jenkinson et al., 2002), spatial smoothing using a Gaussian kernel and highpass temporal filtering as implemented in FSL as well as slice timing correction using a customized text file.

The analysis is done in three steps. On the first level we analyze each session’s data, i.e., execute time-series analysis of the raw 4D fMRI data. We generate voxel-wise parameter estimates of the hemodynamic (blood-oxygen-level-dependent) responses to the different stimuli we used in the fMRI experiment. These voxel-wise parameter estimates represent the change in the blood-oxygenation level for a given stimulus compared to the baseline neural activation of no stimulus presentation and control stimulus. Modeled regressors include cooperation, i.e., choosing up (U) in PD game both in the human and computer conditions, C_PG1 and C_CG1, respectively; defection, D_PG1 and D_CG1; inequity aversion [down (D) in WG], IA_PG2 and IA_CG2; and inequity tolerance, IT_PG2 and IT_CG2. Each explanatory variable is created by convolving the stimulus actual duration times (from onset of stimulus till response using one of the buttons) within each stimulus with a standard gamma hemodynamic response function using FEAT. Through first-level analysis, we obtain parameter estimates as well as statistical maps for each regressor.

On the second level we combine each subject’s activation across several blocks and create contrasts (for human vs. computer conditions: C_PG1 vs. C_CG1, D_PG1 vs. D_CG1, IA_PG2 vs. IA_CG2, IT_PG2 vs. IT_CG2; and WF vs. PD: IA_PG2 vs. C_PG1) using a fixed effects analysis with cluster-level statistical threshold of *Z* > 2.3 and *p* < 0.05. In order to compare human condition to computer condition and inequity aversion in WG to cooperation in PD we subtract one stimulus type (e.g., in Low sociality condition) from another type (e.g., in High sociality condition). The hypothesis of interest here is whether in each voxel the activation to human condition stimuli is greater than in computer condition. We also implement this type of contrast in the opposite direction, i.e., where activation in computer condition is higher than activation in human condition. In result, we generate statistical maps for each of the five contrasts for each subject. These contrast activation maps are registered to each subject’s own high-resolution structural image and also to the Montreal Neurological Institute (MNI) 152-standard template.

Finally on the third level, we use FLAME (FMRIB’s Local Analysis of Mixed Effects) modeling and one-sample *t*-test to decide whether the group activates on average. Mixed effects model the subject variability and, therefore, allow making inferences about the wider population from which the subjects are drawn. Each of the contrasts for the group are Gaussianized intro *Z*-statistical images and thresholded at *Z* > 2.3 with a cluster-corrected significance threshold of *p* < 0.05 (Worsley, 2001). The high resolution structural MRI images of individual subjects are standardized to the MNI space and averaged within the group to create an average structural template.

#### SPM 12 Procedures

The Main Study is analyzed using identical procedures in SPM12 (Wellcome Department of Cognitive Neurology, London, UK^3^), which includes correction for field inhomogeneities, realignment, and coregistration of functional images to each subject’s own high-resolution structural image using a six-parameter rigid body transformation model, reorientation to the plane containing the anterior and posterior commissures, spatial normalization into space compatible with an MNI atlas, and smoothing using a 6 mm^3^ FWHM Gaussian kernel. Statistical analyses are implemented in SPM12. For each participant, event-related condition effects are estimated according to the general linear model, using a canonical hemodynamic response function, high-pass filtering (128 s), and a first-order autoregressive error structure. At the individual level, BOLD signal is modeled in a fixed effects analysis with separate regressors modeling each condition of interest during the game presentation period, for the decision making, and feedback periods. Linear contrasts are created for each comparison of interest (e.g., PD + High Sociality vs. UG + High Sociality, PD + Low Sociality vs. UG + Low Sociality, PD + High Sociality vs. PD + Low Sociality, and UG + High Sociality vs. UG + Low Sociality). These contrasts are then imported to group-level random effects analyses for inference to the population. The above-threshold activations table (shown at *P* < 0.05, FWE) is created with WFU_pickatlas^4^. The particular regions reported in the results are visualized using xjView toolbox^5^.

#### PPI in AFNI

Psychophysiological interaction (PPI) analysis is conducted in AFNI^6^. We define the seed region as medial PFC (defined using the Harvard–Oxford structural atlas^7^). The PPI analysis identifies regions showing differential coupling with the mPFC during High Sociality vs. Low Sociality conditions. The mPFC ROI is projected from MNI space to individual subject space, the time series data are extracted from the combined left and right mPFC, and terms associated with the baseline, linear drift, and head motion are removed. These cleaned time series data are deconvolved with an assumed gamma impulse response function, and then multiplied by the High Sociality vs. Low Sociality condition contrast to generate an interaction term. An additional GLM is implemented as before, but with additional regressors corresponding to the deconvolved mPFC time series, the High Sociality vs. Low Sociality condition contrast, and the interaction of these two regressors, which is the key term in the PPI analysis. This final interaction regressor is used to identify brain regions in which functional coupling with the mPFC differs during the interaction with an Acquaintance compared to Random Individual. Beta weights corresponding to this interaction regressor are converted to *Z*-scores to allow for between-subject comparison.

## Results

### Behavioral Results

In the PD the trend is typical, with high (moderate) levels of cooperation in the first few rounds devolving into consistent moderate (high) levels of defection. The only difference is witnessed between high sociality (humans, acquaintances) and low sociality (computers, random individuals) conditions (**Table 2**). Playing acquaintances in the Main Study resulted in slightly higher levels of cooperation than those in the Pilot Study for Humans condition. Nevertheless, we observe significant difference between cooperation rates in the PD in the Pilot Study (Humans vs. Computers, *N* = 30, *p*-value = 0.002, *t*-test) and in the Main Study (Acquaintances vs. Random Individuals, *N* = 45, *p*-value = 0.0006, *t*-test).

**Table 2:** Computer experiment behavioral results.

Pilot study conditions	Cooperation in PD	Inequity tolerance in WG	Main study conditions	Cooperation in PD	Inequity tolerance in UG
Human	28%	30%	Acquaintance	49%	76%
Computer	16%	46%	Random individual	18%	66%

*Behavioral results of computer experiments for Pilot and Main studies are reported. The percentages of cooperative decisions (choose U or L in the PD) and the frequency of inequity tolerance decisions (choose U in the WG or accepted an unfair offer in the UG) across studies and conditions are listed.*

Results of the Computer Experiment for Welfare and UGs are puzzling. Whereas for WG the egalitarian outcome (inequity aversion), rather than Nash equilibrium (2; 6) is more likely, for the UG accepting unfair offers (inequity tolerance) is predominant. The difference between sociality conditions is not significant.

For the fMRI portion of the experiment the same pattern remains: rates of cooperation are significantly higher for the high sociality condition than those for the low sociality condition (**Table 3**; Pilot Study: Humans vs. Computers, *N* = 10, *p*-value = 0.001, *t*-test; Main Study: Acquaintances vs. Random Individuals, *N* = 15, *p*-value = 0.02, *t*-test). It is important to notice that the cooperation level averages oscillated around the mean till the very last round due to the persistent tests of cooperative strategy by participants that face nasty Nash equilibrium computerized strategy.

**Table 3:** fMRI experiment behavioral results.

Pilot study conditions	Cooperation in PD	Inequity tolerance in WG	Main study conditions	Cooperation in PD	Inequity tolerance in UG
Human	26%	40%	Acquaintance	34%	71%
Computer	15%	62%	Random individual	21%	51%

*Behavioral results of fMRI experiments for Pilot and Main studies are reported.*

We observe significant difference between sociality conditions, for the Welfare and UG (Pilot Study: Humans vs. Computers, *N* = 10, *p*-value = 0.05, *t*-test; Main Study: Acquaintances vs. Random Individuals, *N* = 15, *p*-value = 0.05, *t*-test). The directions of the effect are distinct: acceptance of unfair offers is higher for Computers (low sociality) compared to Humans (high sociality) conditions, but is lower for Random Individual (low sociality) compared to Acquaintance (high sociality) conditions, similar to what is seen in the Computer Experiment. The WG is a simultaneous game, whereas the UG is sequential. In the UG the subject accepts or rejects the offer that is presented to her. In the WG she does not know what will be offered, so it is not necessarily inequity aversion, but might be as well risk aversion. In other words in WG the subject provides a hedge against potential inequity by forcing the egalitarian outcome, whereas in the UG it is not possible without losing everything. That is why in UG most of the subjects tolerate inequity.

### fMRI Results, Pilot Study

The fMRI analysis of the Pilot Study in FSL focuses on the functional activity pattern associated with social domain and economic games participants play. We determine areas in the brain where the neural activation is higher for subjects playing with humans, than playing with computers in completing several different tasks. We report functional activation in the areas as specified in MNI structural atlas (Mazziotta et al., 2001; Collins et al., 2004) and Talairach Daemon Labels atlas (Lancaster et al., 2000).

Cooperation in the PD game with humans compared to cooperation in PD with computers is associated with a signal increase in dorsolateral prefrontal cortex (DLPFC), Brodmann areas (BA) 8 and 9 (Cooperation contrast: BA 8 [*x* = 69, *y* = 69, *z* = 55 (MNI_152 space coordinates)], *Z*-score = 3.01467). Whereas BA 9 functions include sustaining attention and working memory, BA 8 is even more intriguing, as it is linked to the management of uncertainty (Platt and Huettel, 2008) as well as hopes or high expectations. Many studies see DLPFC as a contributor to rational decision-making in social situations. Although cooperation in the PD game is seen by many as irrational, the theory of sociality (Lukinova et al., 2014; Berkman et al., 2015) provides a rational explanation for such behavior by adding an economic component to the subject’s utility function in the social context. Thus, activation in the Cooperation contrast can be attributed to another demonstration of sociality at work, where brain processes cooperation as a rational decision.

Contrast between WG and PD game displays highlight in BA 30 [*x* = 36, *y* = 45, *z* = 33 (MNI_152 space coordinates), *Z*-score = 2.55405] that along with adjacent areas forms posterior cingulate gyrus. Its functions include spatial memory and orientation (Owen et al., 1996), as well as face recognition (Leube et al., 2001). Neither the former, nor the latter directly correspond to the stimuli presented to the subjects. BA 39 [*x* = 61, *y* = 33, *z* = 51 (MNI_152 space coordinates), *Z*-score = 2.54531], located at the middle temporal gyrus, is also involved in calculation (Grabner et al., 2007), as well as in “theory of mind” (Goel et al., 1995), i.e., modeling knowledge, rationality, etc., of another person’s mind. Indeed, calculation and “theory of mind” occur in both games (Rilling et al., 2004), however, while the PD game is familiar and is frequently used in multiple courses in college, the WG, as a rare simultaneous version of UG, requires participants to think through other subject’s strategy and execute the cost-benefit analysis.

### fMRI Results, Main Study

The analysis of neuroimaging data in SPM focuses on the functional activity pattern between games and opponent conditions (**Table 4**; shown at *P* < 0.05, FWE).

**Table 4:** Above-threshold activations (shown at *P* < 0.05, FWE) are presented for the contrasts of interest.

Contrast	Anatomical region	Coordinates (*x, y, z*)	*T*-statistic	*Z*-statistic	Cluster size
**PD > UG**					
	L inferior frontal gyrus	(-38, 3, 34)	11.49	4.87	2
	L inferior parietal lobule	(-41, -44, 58)	10.5	4.72	1
**UG > PD**					
	R superior frontal gyrus	(21, 49, -18)	12.49	4.8	2
**High sociality > Low sociality**					
	L sub-gyral	(-35, -38, 38)	11.08	4.81	1
	L superior frontal gyrus	(-10, 12, 58)	10.63	4.74	1
	R inferior semi-lunar lobule	(12, -60, -52)	9.28	5.15	6
	L pre-central gyrus	(-36, -12, 58)	8.87	5.07	10
	L post-central gyrus	(-40, -36, 64)	8.81	5.05	7
	R cingulate gyrus	(-42, -24, 40)	8.27	4.91	3
	R superior temporal gyrus	(50, 20, -18)	7.87	4.79	1
**Low sociality > High sociality**					
	L inferior parietal lobule	(-35, -47, 46)	13.66	5.16	6
	L inferior parietal lobule	(-35, -56, 46)	13.16	5.09	4
	L inferior frontal gyrus	(-50, 9, 34)	13	5.07	2
	L precuneus	(-7, -62, 54)	11.02	4.8	1
	R precuneus	(3, -56, 54)	10.94	4.79	2
	R cingulate gyrus	(18, -38, 38)	8.83	5.06	1
	R parahippocampal gyrus	(38, -22, -18)	8.58	4.99	1

*MNI_152 space coordinates are reported.*

One of the goals is to determine brain areas where neural activation is higher in one game or another. In Pilot Study, when participant plays in a PD Game compared to her making decisions in a WG besides significant clusters already identified, activation is higher in vmPFC, Brodmann area 32 (BA 32, *x* = 5.70, *y* = 25.53, *z* = 34.81; *T*-statistic = 6.0101). It is likely that brain activation differences between games, namely WG and UGs, are mainly due to calculation issues and the novelty of WG to college students. It is vital that BA 32 associated with rational thought processes does not appear to be more activated, when comparing PD and UG in the Main Study.

When comparing cooperation decision in PD to acceptance of unfair offers in the UG or agreement to Nash equilibrium in the WG, no significant clusters are found. We assert that in the social setting the neural basis for tolerability to defection (opponent’s strategy in PD is nasty) and tolerability to inequity (advantageous position of column chooser in WG and unfair offer in UG) is identical, representing confrontation with the social world that is at times unjust and rough.

**FIGURE 2:**
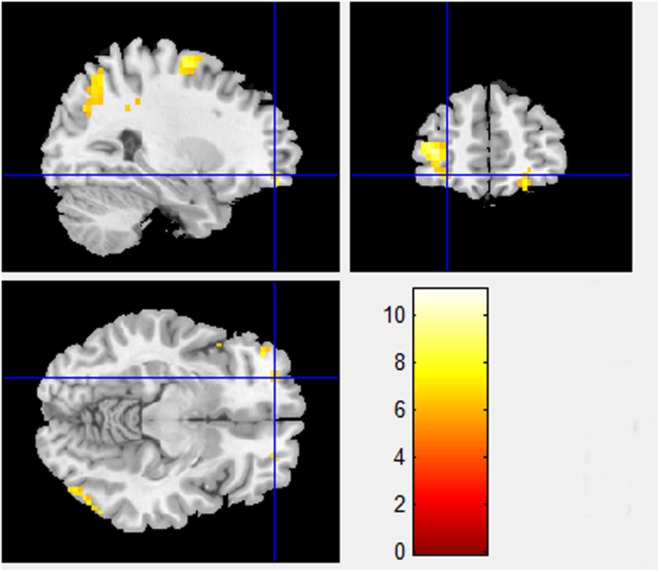
**High sociality > Low sociality contrast (BA 11).** Contrast between High sociality and low sociality conditions reveals activations in orbitofrontal area, BA 11 (*x* = -28.80, *y* = 50.61, *z* = -9.89). This area is associated with planning, reasoning, and decision making in general.

Besides the BA 8 and BA 9 activations already identified in the Pilot Study differences between sociality conditions in PD also show higher activations in orbitofrontal area, BA 11 (*x* = -28.80, *y* = 50.61, *z* = -9.89, *T*-statistic = 6.0101; **Figure 2**), known for its connection to planning, reasoning, and decision making. Cooperation in high sociality condition is indeed attributed to a well-planned and a reasonable, if not rational decision. The contrasts between High sociality and Low sociality involve among others the following brain activations: Superior Frontal Gyri, Inferior Frontal Gyrus, Anterior Cingulate, and Parahippocampal Gyrus.

**FIGURE 3:**
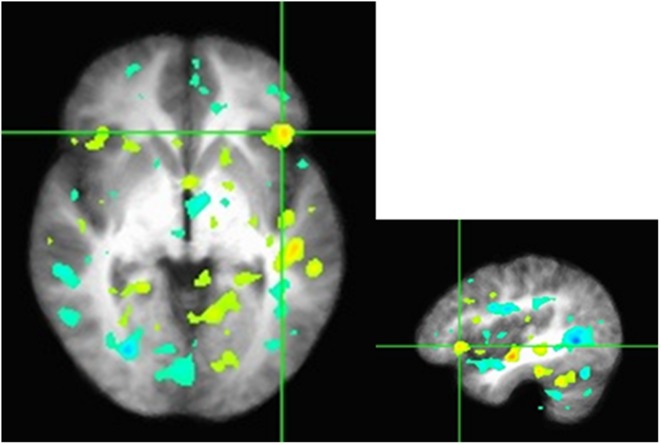
***Z*-scores from the seed to the inferior frontal gyrus in the axial and sagittal views.** Functional connectivity of mPFC with the right inferior frontal gyrus, a region previously associated with cognitive control and modulation of the valuation system. Regions depicted are found to correlate with mPFC to a greater degree when participants make economic decisions as they interacted with others in the high sociality condition compared to the low sociality condition.

We conduct preliminary connectivity tests using PPI analysis using the mPFC as a seed, a brain region associated with valuation. The software used for obtaining the activations is Analysis of Functional Neuro-Images (AFNI). The presence of a positive context-dependent interaction in a region (i.e., a PPI) can be interpreted as greater relative connectivity between that region and the seed in one condition compared to another. In this case, the regions depicted in **Figure 3** are found to correlate with mPFC to a greater degree when participants made decision in PD as they interacted with others in the high sociality condition compared to the low sociality condition. **Table 5** shows the correlation coefficients for the areas of interest, i.e., the areas highlighted for the High > Low Sociality contrast.

**Table 5:** R coefficients with seeded ROI in the medial frontal gyrus (coefficient range -0.0019445 to 0.01676).

	AFNI-r coefficient	# of voxels	% max coefficient
Fusiform gyrus	0.006777	1492	0.4043
Inferior frontal gyrus	0.006322	731	0.3772
Insula	0.006274	509	0.3743
Anterior cingulate	0.006351	381	0.3789
Parahippocampal gyrus	0.005702	306	0.3402

*Regions that were significantly positively correlated with medial prefrontal cortex (mPFC) to a greater degree when participants made economic decisions as they interacted with others in the high sociality condition compared to the low sociality condition: r refers to the Pearson correlation coefficient relating neural activity in that cluster and mPFC activation coefficients at the group level.*

Several regions emerge that have previously been implicated in prosocial economic choice (e.g., the right inferior frontal gyrus and the DLPFC; Tabibnia et al., 2008). Notably, these regions modulate activation of the valuation system in cases when self-control is necessary to override impulsive or habitual choices such as selfish economic decisions (Hare et al., 2008). Several small peaks also emerge in the valuation network proper (not shown), but the small sample size prevents strong inferences based on these data.

## Discussion

If social cognition constantly results in a different pattern of brain activity than a non-social one and the regions of brain activation during social cognition have a special status (high levels of activity even at rest) in the brain (Adolphs, 2003; Jenkins and Mitchell, 2011), to what extent are brain systems that control social behavior domain specific (Cosmides and Tooby, 1994; Stone et al., 2007)? If evolutionary perspective provides theoretical grounding for domain specificity, neuroscience then gives an opportunity to investigate it.

This paper focuses on the comparison of two sociality conditions. It is the first attempt to find what brain regions correspond to a neural value of sociality and lay a foundation to identify and estimate this neural value. The key is to induce prosocial behaviors in economic games, different in nature, but common in representation. Our findings support the theory of sociality. Indeed, we assert that additional social utility is calculated in the human brain, when a person interacts with someone from the socialized group she identifies herself with. This additional social utility may cause prosocial decisions, such as cooperation in the PD, and may as well appear rational to the brain. A good way of talking about sociality is by illustrating what happens when it is impaired (Edmiston et al., 2015): sociality can be described as the opposite of autism.

Based on our research we propose that economic games, such as PD representing collective action and the UG, the simplest demonstration of bargaining, can be intertwined. We observed that presenting UG and PD together results in higher cooperation rates than when participants deal with the PD only. One explanation is a spillover effect produced during the UG. In the social environment, norms of fairness in the UG may encourage us to be as well fair in the PD, i.e., cooperate. Recent findings, however, do not report any correlation between rejecting unfair offers and prosocial behaviors in other games (Yamagishi et al., 2012). The fact that we did not observe significant differences in activations between the games could be due to highly perplexed cognitive processes involved in these social dilemmas when sociality is induced.

The data collected from the fMRI experiments can serve to answer many more questions, than those raised in this paper. For example, how do subjects perceive outcomes? While in this study, our main focus is on the stimuli from onset till response, next we can take into account the reaction toward the outcome displayed to the participant. How will participants react to unfairness or defection? Or how will participants react and what brain activation will be related to it if they defect, while the opponent cooperated? Why do dopaminergic and subcortical regions show no activation in the task? These research questions: to identify the brain regions that are sensitive to sociality and to test the relative effectiveness of the sociality inductions in altering neural activation in the social cognition and valuation networks and economic decisions – hold promise to advance the social aspects of BCI (Sexton, 2015).

## Author Contributions

All authors designed the experiments; EL carried out the experiments, programmed the software for the experiment, prepared the data, and conducted the analysis; EL wrote the paper; all authors reviewed the paper and the results.

## Conflict of Interest Statement

The authors declare that the research was conducted in the absence of any commercial or financial relationships that could be construed as a potential conflict of interest.

## Footnotes

1 www.neurobehavioralsystems.com

2 www.fmrib.ox.ac.uk/fsl

3 www.fil.ion.ucl.ac.uk/spm/

4 http://www.nitrc.org/projects/wfu_pickatlas/

5 http://www.alivelearn.net/xjview

6 http://afni.nimh.nih.gov/sscc/gangc/CD-CorrAna.html

7 http://www.cma.mgh.harvard.edu/
